# Structure of a Diguanylate Cyclase from *Thermotoga maritima*: Insights into Activation, Feedback Inhibition and Thermostability

**DOI:** 10.1371/journal.pone.0110912

**Published:** 2014-10-31

**Authors:** Angeline Deepthi, Chong Wai Liew, Zhao-Xun Liang, Kunchithapadam Swaminathan, Julien Lescar

**Affiliations:** 1 Department of Biological Sciences, National University of Singapore, Singapore, Singapore; 2 School of Biological Sciences, Nanyang Technological University, Singapore, Singapore; 3 Centre d' Immunologie et des Maladies Infectieuses, Centre Hospitalier Universitaire Pitié-Salpêtrière Faculté de Médecine Pierre et Marie Curie, Paris, France; University of Cantebury, New Zealand

## Abstract

Large-scale production of bis-3′-5′-cyclic-di-GMP (c-di-GMP) would facilitate biological studies of numerous bacterial signaling pathways and phenotypes controlled by this second messenger molecule, such as virulence and biofilm formation. C-di-GMP constitutes also a potentially interesting molecule as a vaccine adjuvant. Even though chemical synthesis of c-di-GMP can be done, the yields are incompatible with mass-production. tDGC, a stand-alone diguanylate cyclase (DGC or GGDEF domain) from *Thermotoga maritima*, enables the robust enzymatic production of large quantities of c-di-GMP. To understand the structural correlates of tDGC thermostability, its catalytic mechanism and feedback inhibition, we determined structures of an active-like dimeric conformation with both active (A) sites facing each other and of an inactive dimeric conformation, locked by c-di-GMP bound at the inhibitory (I) site. We also report the structure of a single mutant of tDGC, with the R158A mutation at the I-site, abolishing product inhibition and unproductive dimerization. A comparison with structurally characterized DGC homologues from mesophiles reveals the presence of a higher number of salt bridges in the hyperthermophile enzyme tDGC. Denaturation experiments of mutants disrupting in turn each of the salt bridges unique to tDGC identified three salt-bridges critical to confer thermostability.

## Introduction

Bis-(3′-5′) cyclic diguanosine monophosphate (c-di-GMP) is a second messenger that was initially discovered as an allosteric activator of cellulose synthase in *Gluconobacter xylinus* [1]. Binding of c-di-GMP to various effector or receptor proteins regulates the expression of genes controlling the motility, virulence and biofilm formation of several bacterial species [2]–[4]. Cellular levels of c-di-GMP are tightly regulated by the opposing activities of diguanylate cyclases (DGCs) [5]–[7] that synthesize c-di-GMP from two GTP molecules and phosphodiesterases (PDEs) [8], [9] that hydrolyze c-di-GMP into 5′-phosphoguanylyl- (3′-5′) - guanosine (pGpG) and GMP. A large number of genes bearing the PDE or DGC sequence signature motifs were identified in bacterial genomes, indicating a prominent role of c-di-GMP signaling pathways in regulating their community behavior [10].

Catalytically active DGCs bear the GG(D/E)EF consensus amino-acid sequence at their active site (A-site) and a conserved RxxD motif at an inhibitory site (I-site) [11]. The I-site binds to the product and mediates feedback inhibition. The A- and I-sites are located at opposite ends of the GGDEF domain [11]. Crystal structures of several proteins containing DGC domains have been reported, such as PleD [5] from *Caulobacter crescentus*, DgcZ [12] from *Escherichia coli* K-12 and WspR [6], [13] from *Pseudomonas aeruginosa* and *Pseudomonas syringae*. Isolated GGDEF domains structurally characterized include YfiN from *Pseudomonas aeruginosa* [14] and Xcc4471 from *Xanthomonas campestris* [15]. C-di-GMP synthesis from two separate GTP molecules requires dimerization of two active GGDEF domains, with the loops bearing the GGDEF motifs of each monomer brought in close proximity to initiate the cyclization reaction. Several proteins containing a DGC catalytic domain also possess an N-terminal domain such as PAS, GAF, CheY and HAMP, that control their diguanylate cyclase activity in response to upstream activating signals, often as part of two-component signaling systems [16]. Thus, the N-terminal domain of PleD plays a crucial role in orienting the two GGDEF domains in either a productive dimeric conformation for c-di-GMP synthesis or in a non-productive arrangement of the two half active sites, to prevent catalysis [7]. A similar mechanism is observed in DgcZ from *E. coli* where binding of Zinc to the N-terminal CZB domain induces an inactive dimeric arrangement of the GGDEF domains [12]. In the case of WspR, inhibition proceeds through the formation of different oligomeric states mediated by a helical stalk domain^4^. Thus, two mechanisms can chiefly account for the feedback inhibition of DGCs mediated by the c-di-GMP product: allostery [11] and domain immobilization [7]. According to the allosteric model, binding of c-di-GMP to the enzyme I-site triggers structural changes across the protein, leading to a rearrangement of the A-site residues into an inactive conformation. In contrast, inhibition could proceed from the bridging action of c-di-GMP, which, when bound to the I-site, locks two GGDEF domains in a catalytically incompetent state, where the two A sites of the dimer are maintained apart from each other. This was observed in PleD, where an inactive dimer is stabilized by a bound c-di-GMP molecule on both monomers at the primary and secondary I-sites [17]. A similar inactive dimer was also observed for a GGDEF domain from *Marinobacter aquaeoli* [18].

Large-scale production of c-di-GMP would facilitate biological studies of several bacterial signaling pathways and phenotypes controlled by this molecule. Moreover, the immunomodulatory properties of c-di-GMP open up the possibility of using it as a vaccine adjuvant [19], [20]. Chemical synthesis of c-di-GMP was achieved, but yields are not economical [21]. Recently, Rao *et al.* [22] cloned and expressed a GGDEF domain from *Thermotoga maritima,* hereafter named tDGC, that enables robust enzymatic production of large quantities of c-di-GMP. Enzyme assays showed that tDGC is active even after days of incubation at 30°C but with very low product production, irrespective of substrate concentration, due to feedback inhibition. However, a mutation (R158A) at the I-site of tDGC significantly alleviates production inhibition, enabling synthesis of several hundred milligrams of c-di-GMP with just 10 mg of the mutant enzyme [22].

In order to understand the structural determinants of tDGC thermostability and to investigate the catalytic mechanism and feedback inhibition of this biotechnologically important enzyme, we crystallized tDGC (i) in an active-like conformation with two A-sites in proximity, and (ii) in an inactive conformation, with c-di-GMP bound to the I-site. A comparison between these two structures enables us to examine potential conformational changes propagated between the I- and A- site upon c-diGMP binding to the I-site. We also report the structure of the R158A mutant of tDGC. Structural comparison with DGC homologues from mesophilic bacteria reveals the presence of a higher number of salt bridges in tDGC. Using circular dichroism (CD), three key salt bridges responsible for the enhanced thermostability of tDGC were identified.

## Materials and Methods

### Protein expression and purification

The gene encoding the complete (248 amino-acids) wild type protein TM1788 (Uniprot: Q9X2A8) from *Thermotoga maritima*, with codons optimized for bacterial expression, was purchased from Genscript. The gene corresponding to residues 82–248 (comprising the GGDEF domain, referred to as “tDGC protein”) was cloned into the pET28b vector between *Nde1* and *Xho1* restriction sites). The R158A feedback inhibition null mutant was purchased from Genscript. All proteins containing six Histidine residues at their N-terminus were overexpressed in *E. coli* BL21 (DE3) cells by the addition of 0.8 mM IPTG at 37°C for 4 hours. The tDGC protein was initially purified by immobilized metal affinity chromatography (IMAC) using Ni-NTA agarose (Qiagen) and further purified by ion exchange using a HiTrap column (GE Lifesciences) and size exclusion on a Superdex 75 column (GE Lifesciences). The RocR PDE was expressed and purified as described [23]. Nucleotide-free tDGC protein was obtained by treatment with RocR in the presence of 5 mM MgCl_2_. The mixture of RocR, tDGC and pGpG was separated by gel filtration. The identity of the eluted pGpG was confirmed by mass spectrometry.

### Protein crystallization

Purified tDGC was concentrated to 10 mg/ml and its homogeneity assessed by dynamic light scattering on a DynaPro instrument. Initial crystallization screening was performed by sitting-drop vapor diffusion at 18°C, using commercial crystallization screens from Hampton Research (Crystal Screens 1 and 2, PEG/Ion, Index and SaltRx). Intelli 96–3 wells sitting drop plates were used to test three precipitant:protein ratios (1∶1, 1∶2 and 2∶1) using a Phoenix crystallization robot (Art Robbins Instruments). Crystals of the inactive dimer were obtained with 0.1 M monobasic sodium phosphate monohydrate, 0.1 M monobasic potassium phosphate, 0.1 M MES monohydrate (pH 6.5), 2 M NaCl. The R158A mutant was crystallized with 2 M NaCl, 10% (w/v) PEG 6000. The active-like dimer was crystallized with 0.15 M lithium sulfate, 0.1 M citric acid (pH 4.0), 10% (w/v) PEG 6000. The protein used to obtain the latter crystal form was also the c-di-GMP-locked inactive dimer. However, upon mixing with precipitant, the crystallization drops turn milky white, indicating protein precipitation. Upon overnight incubation at 18°C, the crystallization drops become clear again and crystal formation is observed after 4–5 days. Thus, the active-like dimer is likely to originate from a rearrangement following initial protein precipitation.

The diffraction properties of these crystals were improved by overnight soaking in a precipitating solution containing 14% (w/v) PEG 6000 prior to flash-freezing in liquid nitrogen, extending the diffraction limits from 3.5 Å to 1.9 Å.

### Data collection and structure solution

X-ray diffraction data were collected at SLS (Vilingen, Switzerland) and processed using iMosflm [24]. The structure was determined by molecular replacement using the BALBES [25] server with the DGC structure from *Methylococcus capcsulatus* (PDB code: 3ICL), which has 40% amino-acid sequence identity with tDGC, as a search probe. The structure was built by iterative cycles of model building at the computer graphics using COOT [26] and refinement using Autobuster [27]. The geometrical parameters for c-di-GMP were generated using the c-di-GMP coordinates 2V0N from the PDB (www.rcsb.org). Buried solvent accessible surface areas upon dimer formation were calculated using the PDBePISA server [28]. The quality of the structures was assessed using the MOLPROBITY [29] server and the figures were generated using the Pymol software [30]. Data collection and structure refinement parameters are summarized in **Tables 1** **and** **2**. The atomic coordinates and structure factors are deposited with the Protein Data Bank under accession codes 4URS (for the c-di-GMP bound inactive dimer), 4URG (for the c-di-GMP bound active-like dimer) and 4URQ (for the apo R158A mutant).

**Table 1:** Data collection statistics.

tDGC	c-di-GMP bound inactive dimer	c-di-GMP bound active-like dimer	c-di-GMP free R158A
Detector Type	Pilatus	Pilatus	MAR CCD
Synchrotron	SLS PXIII	SLS PXIII	NSRRC 13B1
Wavelength (Å)	1.00	1.00	1.00
Resolution (Å)	32.57–2.27 (2.45–2.27)	36.49–1.9 (36.49–1.9)	38.38–2.5 (2.56–2.5)
Space Group	P1	C2	C2
Molecules per asymmetric unit	2	2	6
Cell parameters (Å)	37.15, 37.14, 62.28	108.89, 52.12, 73.71	159.41 91.98 87.59
α/β/γ (°)	95.48, 100.23, 107.78	90, 124.05, 90	90, 90.04, 90
Measured reflections a	62,597 (8,725)	184,283 (27,210)	323,030 (47,601)
Unique reflections a	13,630 (1,971)	27,197 (3,951)	43,759 (6,374)
Redundancy	4.6 (4.4)	6.8 (6.9)	7.4 (7.5)
I/σ a	6.5 (2.6)	11.4 (5.9)	15.5 (3.0)
Completeness (%)^a^	99.6 (99.5)	100 (100)	99.7 (100)
R_merge_ a	0.143 (0.508)	0.118 (0.325)	0.088 (0.694)

a The values for the highest resolution shell are shown in parenthesis.

**Table 2:** Refinement statistics.

Tdgc	c-di-GMP bound inactive dimer	c-di-GMP bound active-like dimer	c-di-GMP free R158A
Reflections used for refinement	13,625	27,172	43,743
Non-hydrogen atoms	2,980	2764	7531
Water	93	144	127
R_work_ (%)	21.80 (19.50)	20.73 (20.79)	22.20 (23.21)
R_free_ (%)	26.80 (27.13)	24.13 (21.09)	24.67 (25.43)
r.m.s.d. bond lengths (Å)	0.013	0.014	0.014
r.m.s.d. bond angles (°)	1.71	1.47	1.63
Mean B-factor (Å^2^)			
Protein	38.8	25.2	53.3
Ligands:			
C-di-GMP	45.6	10.2	
Glycerol	47.5		
MES	63.2		43.4
Water	38.9	31.1	
Ramachandran Plot			
Most favored (%)	98.42	98.69	95.72
Allowed(%)	1.58	1.31	4.17
Outliers (%)	0.99	0	0.11
PDB code	4URS	4URG	4URQ

### Site-directed mutagenesis

For thermostability analysis, point mutations were introduced into the wild type tDGC GGDEF domain in the pET22b vector. The forward primers: 5′ ACC ATT CTG CTG TAT GCC ATG AAA GAA GAA TAT 3′ (for D177A; mutant codon underlined), 5′ CTG AGC ACC TTT CGT GCC CCG GTG CGT GTG GAA 3′ (for E196A) and 5′ GAA AGT GGC GGA TAT GGC TAT GTA TAA AGC CAA AG 3′ (for R233A) along with the respective complementary primers were used. The mutations were confirmed by sequencing and the constructs were overexpressed and purified following the same protocol that was used for the wild type tDGC protein.

### Melting temperature assay

Circular dichroism (CD) measurements were performed using a Jasco spectropolarimeter (J-810) with a 1 mm path length quartz cuvette. To determine the melting temperature, 200 µl protein samples at a concentration of 0.8 mg/ml, in 20 mM Tris-HCl (pH 8.5), 250 mM NaCl, 5% (v/v) glycerol, 10 mM MgCl_2_ were used. The temperature was gradually increased from 25 to 95°C at the rate of 1°C/min and ellipticity was recorded at a wavelength of 220 nm for every 0.2°C increment in temperature. The melting temperature was determined by plotting the CD data at 220 nm against temperature and fitting to a Boltzmann sigmoidal curve (between 60 and 95°C) using the curve fitting software XL Fit4.

## Results

### Monomer structure

**Figure 1:**
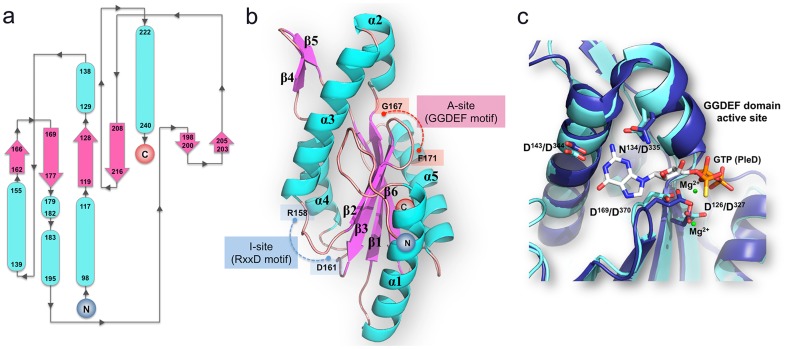
Domain architecture of tDGC. (**a**) Topology of the GGDEF domain of tDGC. The N- and C- termini of the polypeptide chain are indicated. The β-strands are depicted by pink arrows and α-helices by blue tubes. (**b**) Location of the A- and I-site on the structure of tDGC: The loops bearing the “GGDEF” motif at the A-site and “RxxD” motif at the I-site are shown in red and blue respectively. (**c**) Superposition of the A site of tDGC (cyan) and PleD (dark blue)^7^. GTPαS-Mg^2+^, bound to PleD is shown as sticks and green spheres respectively. Residues involved in metal ion binding and base recognition (represented as sticks and labeled according to tDGC and PleD numbering schemes) are strictly conserved between the two proteins.

We expressed and crystallized a domain of the TM1788 protein from *Thermotoga maritima* (248 residues, WP_010865403) spanning residues 82–248, hereafter named tDGC. The structures of tDGC and of its R158A mutant were determined in three crystal forms (i) tDGC in an active-like dimeric conformation (ii) tDGC in an inactive dimeric conformation (iii) R158A mutant in a monomeric conformation (**Tables 1** **and** **2**). The overall structure of the tDGC monomer features a central antiparallel β-sheet with the topology β2-β3-β1-β6, surrounded by five α-helices (**Fig. 1a** **)**. The location of the conserved catalytic 167-GGDEF-171 motif (A-site) and the inhibitory I-site (158-RxxD-161) on loops connecting β2 and β3 and between α3 and β2 respectively, are depicted in **Fig. 1b**. tDGC shares a fold similar to adenylyl cyclases and the palm domain of polymerases, with a reaction mechanism involving two metal ions, which are observed in the structure of PleD, crystallized with GTPαS, but not in the present structures. According to this reaction scheme, Asp169 deprotonates the 3′ hydroxyl group of the ribose of the GTP substrate to initiate an in-line nucleophilic attack on the α-phosphate of the target GTP, followed by elimination of the pyrophosphate moiety (**Fig. 1c** **)**. The four residues responsible for coordinating the Mg^2+^ ion (Asp126 and Asp169) and for making contact with the guanine moiety (Asn134 and Asp143) are strictly conserved in tDGC, as shown in **Fig. 1c**.

### Inactive dimer mediated by c-di-GMP bound at the I site

**Figure 2:**
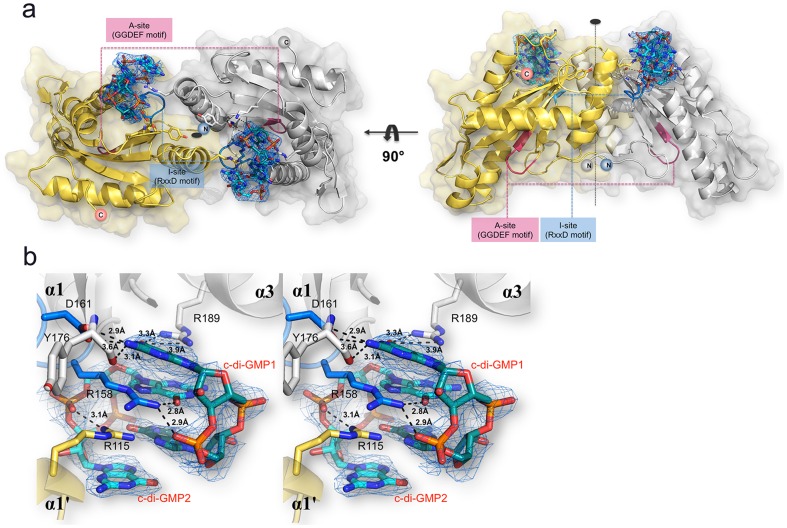
The inactive tDGC dimer (a) The two monomers present in the asymmetric unit are colored in grey and yellow respectively with the two bridging c-di-GMP molecules shown as sticks. The views are along (left) and perpendicular (right) to the non-crystallographic dyad. (**b**) Stereoview of the dimerization mediated by two c-di-GMP molecules (labeled c-di-GMP1 and c-di-GMP2), bound at the I-site of tDGC, forcing the GGDEF domain in an inhibited conformation, with both A-sites facing away from each other. An omit map (blue mesh) with Fourier coefficients 2F_o_-F_c_, where the c-di-GMP ligand was omitted from phase calculation is shown at 1σ contour level. Residues from the RxxD motif at the I-site forming hydrogen bonds with the bound c-di-GMP molecules are displayed as sticks and the distances between interacting atoms are displayed. The same color code as in panel **a** is used.

**Figure 3:**
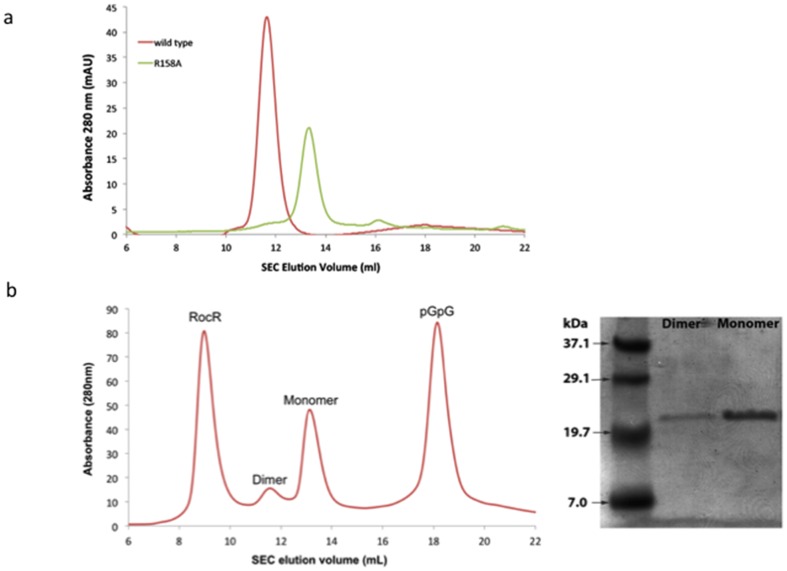
Size Exclusion Chromatograms (a) Gel filtration chromatogram using a S75 column, of the wild type tDGC protein (red) that elutes as a c-di-GMP-bound dimer and of the R158A mutant (green) that purifies as a monomer and is devoid of nucleotides. (b) Following incubation of the wild type tDGC bound to c-di-GMP with RocR (in the gel filtration buffer containing 25 mM Tris-HCl, 300 mM NaCl, 5% (v/v) glycerol, 10 mM MgCl_2_ for 2 hrs at room temperature), c-diGMP is converted to pGpG and tDGC elutes predominantly as a monomer. SDS PAGE analysis of the dimeric and monomeric fractions demonstrates that both peaks contain tDGC.

The wild type tDGC protein co-purifies with c-di-GMP and co-crystallizes without any extraneous addition of ligand. The structure was determined at a resolution of 2.27 Å (**Tables 1** **and** **2**). In this crystal form, tDGC adopts a dimeric conformation, where the two monomers are related by a non-crystallographic dyad (**Fig. 2**). The buried accessible surface area between the two monomers is 1,235 Å^2^ and their interface is stabilized by one salt bridge and thirteen hydrogen bonds. I-site residues 158-RxxD-161 from the two monomers interact with two molecules of c-di-GMP that are mutually intercalated (**Fig. 2a**). This symmetrical arrangement is similar to the PleD structure [7], with the four guanyl bases of the two c-di-GMP molecules bound to the primary inhibitory sites of one molecule comprising the 158-RxxD-161 motif and a secondary inhibitory site R115 contributed by the second monomer. Residue R158 plays a key role in the interaction with both c-di-GMP molecules, via its guanidinium moiety that completes the set of stacking interactions (**Fig. 2b**). In this arrangement, the two monomers are locked in an inactive orientation with their A sites facing away from each other in a catalytically unproductive mode (**Fig. 2a**). The crystallographic dimer observed in this crystal form is consistent with gel filtration analysis showing that tDGC forms a dimer in solution (**Fig. 3a**). To demonstrate that dimerization is mediated by c-di-GMP, which co-purifies with tDGC, we incubated the protein with RocR, a PDE from *Pseudomonas aeruginosa*, to remove the bound c-di-GMP. Remarkably, following enzymatic treatment with RocR, the wild type tDGC protein elutes as a monomer (**Fig. 3b**).

### I-site mutant (R158A) structure

The R158A mutant structure was determined at a resolution of 2.5 Å (**Tables 1** **and** **2**). The protein does not establish large contacts in the crystal packing, which is consistent with its monomeric form in solution (**Fig. 3a**). When superimposed with the wild-type tDGC protein, the r.m.s.d. is 0.99 Å for 152 Cα atoms, suggesting the absence of large conformational changes between the two proteins. The R158A mutation abolishes binding of the c-di-GMP product and eliminates feedback inhibition [22]. From their gel filtration elution profile, the R158A mutant elutes as a monomer, whilst tDGC elutes as a dimer (**Fig. 3a**). The sole mutation R158A is thus able to eliminate the binding of the c-di-GMP ligand to the I- site, restoring the protein to its monomeric form, and preventing feedback inhibition (**Fig. 3**).

### tDGC in active-like dimer conformation

**Figure 4:**
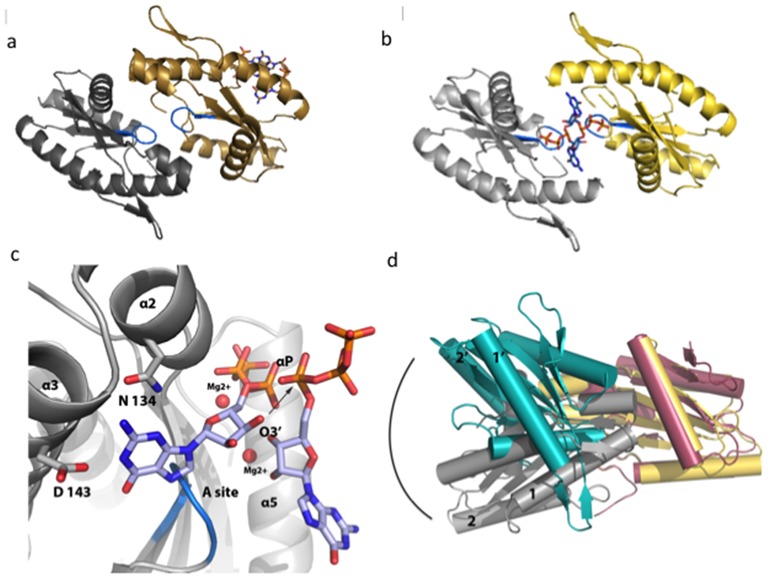
The active-like tDGC dimer and the cyclization reaction. (**a**) Structure of tDGC crystallized in a dimeric active-like conformation, with the two half active sites (loops containing the GGDEF motif colored in blue) facing each other. One monomer is colored yellow and the other grey. The single c-di-GMP molecule bound to the I-site of the monomer colored in yellow, is shown as sticks. (**b**) “Optimized” dimer where the residual transformation (**Figure S2** in File S1) was applied to the monomer colored grey to generate a dimer with exact two-fold symmetry. The view is along the non-crystallographic dyad that runs across the two GTP molecules displayed as sticks. (**c**) Magnified view of the tDGC-GTP-Mg Michaelis complex, modeled on the basis of the “optimized” 2-fold symmetric dimer. The arrows indicate the nucleophilic attack of the 3′ oxygen atom on the α-phosphate of the adjacent GTP. (**d**) Superposition of the “optimized” active dimer (this work, yellow and grey) and the c-diGMP cross-linked YdeH dimer (PDB code: 3TVK, Zaehringer and Schirmer) (colored in pink and teal).

The structure of tDGC at pH 4, in an alternative dimeric form, was determined to a resolution of 1.9 Å (**Tables 1** **and** **2**). In this crystal form, two c-di-GMP molecules are bound to the I-site of a single tDGC monomer and the A-sites are brought close to each other (**Fig. 4a**). The absence of a second c-di-GMP molecule bound to the other monomer of the asymmetric unit, eliminates the locking of the monomers in a non-productive orientation. The buried interface area between the two monomers is 905 Å^2^. In this novel dimeric arrangement, the two monomers are related by a rotation of 167°, a value close to the transformation thought to lead to an active 2-fold symmetric dimer, poised to react with two GTP substrate molecules to perform the cyclization reaction [7] (**Fig. 4a**). According to the postulated mechanism for the formation of c-di-GMP, each A-site binds to a GTP molecule to form a 2-fold symmetric active dimer with the A sites in proximity [7]. Despite several attempts using soaking or cocrystallization, we could not observe bound GTPαS in this crystal form. The crystals were obtained at pH of 4 at which the carboxylic groups of the aspartate residues involved in metal binding and base recognition (Fig. 1c) are likely to be protonated. The rather acidic conditions of crystallization are therefore likely to hamper metal and substrate binding although the protein conformation looks active (Fig. 4). The structure of PleD^7^ bound to GTPαS was therefore used as a guide to position two GTP molecules in an optimized 2-fold symmetric dimer (**Fig. 4b**). Only minor structural movements are then required to positions the α-phosphate groups of the two GTP molecules near the 3′oxygen atoms of the ribose of the adjacent GTP, showing how nucleophilic attack can initiate the cyclization reaction (**Fig. 4c**).

### Comparison of thermostable tDGC with mesophilic homologues

**Figure 5:**
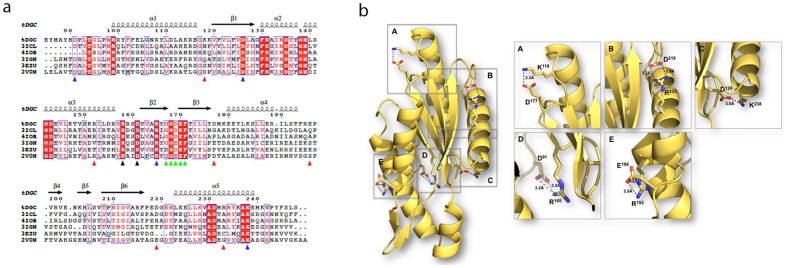
Structure-based sequence alignment of selected DGCs and salt-bridges unique to tDGC. (**a**) Sequence alignment between tDGC from *Thermotoga maritima* (this work), mesophilic DGC domains (3ICL, 4IOB, 3IGN and 3EZU). The sequence of the structurally characterized PleD (2V0N) from *Caulobacter crescentus* is also included for comparison. The secondary structure elements of tDGC are displayed above the sequences. Strictly conserved residues are in red boxes and chemically similar residues are colored in red. Residues D126 and D169 of tDGC are involved in divalent metal coordination. The A- and I-site residues are highlighted with green and black triangles respectively. The blue triangles indicate salt-bridge forming residues that are conserved across the five proteins. Red triangles indicate residues unique to tDGC involved in salt bridge formation. (**b**) Mapping of the salt bridges unique to the thermostable enzyme onto the 3D structure of tDGC: Lys118-Asp177, Arg233-Asp219 and Arg152–Glu196 were disrupted in the site-directed mutagenesis study.

The thermostable GGDEF domain of tDGC shares a conserved structure with homologous proteins from three mesophilic species: *Pseudomonas aeruginosa, Marinobacter aquaeolei* and *Geobacter sulfurreducens* (**Table 1** in File S1) and one thermo-tolerant organism *Methylococcus capsulatus* ^16^. Several molecular factors that potentially contribute to thermostability were examined (**Table 1** in File S1). This comparison revealed that the increased thermostability of tDGC could be predominantly due to an excess of salt bridges (total of 5) compared to mesophilic homologues (1 or 2) (**Table 2** in File S1). Only salt bridges that connect residues far apart in the tDGC amino-acid sequence were considered, as protein unfolding would not disrupt salt bridges involving residues adjacent in the sequence. A sequence alignment (**Fig. 5a**) reveals that of the five salt bridges present, two are conserved across protein homologues (D91-R165 and D126-K238), whilst the three salt bridges: K118-D177, R152-E196 and R233-D219 (**Fig. 5**) are unique to the thermostable enzyme. This is consistent with the observation that several *Thermotoga maritima* proteins possess an excess of salt bridges, compared to their mesophilic counterparts [31], [32].

### Contribution of salt bridges to thermostability

**Figure 6:**
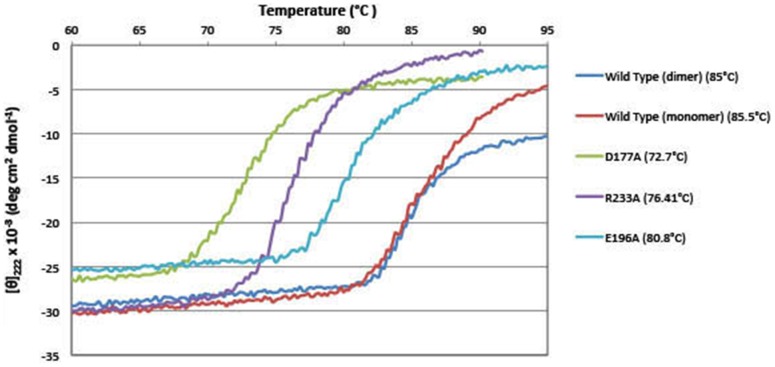
Temperature-dependent unfolding of tDGC and of tDGC single mutants recorded by CD spectropolarimetry. The mean residue molar ellipticity vs temperature for proteins tDGC (dimeric form), tDGC (monomeric form, following treatment with RocR), D177A, E196A and R233A is plotted. A significant difference in the melting profiles of the wild type and the single tDGC mutants (disrupting individual salt bridge mutants) is visible. The melting temperature T_m_, was determined by a Boltzmann sigmoid analysis (see text).

To analyze the contribution of each salt bridge to tDGC thermostability, single mutants D177A, E196A and R233A were made to disrupt the three unique solvent exposed salt-bridges (**Fig. 5b**). The three mutants eluted as dimers in gel filtration, indicating that c-di-GMP is bound to their I-site. To evaluate the stabilization effects brought about by nucleotide binding and dimerization in the thermal denaturation experiments, the nucleotide-free monomeric form of the wild type protein was generated by incubating with RocR [23], in order to digest the c-di-GMP molecule bridging the two tDGC monomers. This digestion step was followed by gel filtration to remove RocR and pGpG. The melting temperatures of the wild type tDGC and the mutants were measured by circular dichroism at 220 nm (**Fig. 6**). The monomeric and dimeric forms of the wild type tDGC protein exhibit comparable melting temperatures with T_m_ values of 85.5°C and 85.0°C respectively. Disruption of the R152-E196 salt bridge has the smallest effect on tDGC thermostability (decrease of 4.2°C). However, disruption of the other two salt bridges, K118-D177 and D219-R233, lead to large reductions in the melting temperature of tDGC by 12.3°C and 8.6°C, respectively (**Fig. 6**).

## Discussion

We report the structure of tDGC from *Thermotoga maritima*, an important enzyme for the large scale enzymatic synthesis of c-di-GMP and the first GGDEF domain from a hyperthermophile. In its inactive form, the tDGC dimer is closely superimposable to the GGDEF dimers from *Marinobacter aquaeolei* (3IGN)[18], PleD from *Caulobacter crescentus* (2V0N)^7^, WspR from *Pseudomonas syringae* (3I5A) [13] and WspR from *Pseudomonas aeruginosa* (3BRE) [6], suggesting a common mechanism of inhibition by domain immobilization. This dimeric form is mediated by two intercalated c-di-GMP molecules bridging the two monomers, such that its two half-active sites are placed in a non-productive orientation. The arginine and aspartate residues of the RxxD primary I-site motif are conserved in 57% of diguanylate cyclases [7]. In addition, 64% of proteins that contain this inhibitory motif have a secondary secondary binding site (R115) on an adjacent subunit that participates in locking the domains together. Thus, this mode of restricting interdomain mobility could constitute a widespread mechanism of regulation and feedback inhibition of GGDEF proteins devoid of other regulatory domains. In the case of tDGC, however, we did not examine possible contributions from its N-terminal 83 residues in regulating the formation of an active or inactive GGDEF dimer.

Based on simulations of bound and free forms of the GGDEF domains, it was previously proposed that product binding at the I-site could induce rearrangements of A-site residues, through a allosteric signal transmitted between these two distant sites [11]. To investigate if c-di-GMP binding at the I-site causes conformational changes at the A-site, the structures of the active-like and inactive dimer were compared (**Figure S1** in File S1). The backbone and side-chain conformations are closely similar, suggesting the absence of signal propagation between the A- and I-sites, upon c-di-GMP binding. We therefore conclude that stand-alone GGDEF domains are inhibited predominantly through domain immobilization mediated by c-di-GMP bound at the I-site.

Using the tDGC active-like dimer as a guide, we were also able to propose a 2-fold symmetrical dimer conducive to GTP cyclization with a nucleophilic attack by the 3′ oxygen groups of the ribose. Further structural and enzymatic studies are now needed to confirm this model.

Although various structural features could account for thermostability, a study that compared 94 proteins structures from the hyperthermophile *Thermotoga maritima* with protein homologues from mesophilic organisms showed an increased number of salt bridges and compactness of the protein [33]. Another study reported that 73% of proteins from *Thermotoga maritima* have a higher “contact order” (average sequence distance between all pairs of contacting residues) compared to their mesophilic counterparts [34]. tDGC shares most structural parameters with its mesophilic counterparts (**Table 1** in File S1), but it has three exclusive salt bridges. From the denaturation assay, the difference in the melting temperature of the wild type protein in its dimeric form and monomeric form are similar (T_m_ ca 85.0°C), indicating that the nucleotide-mediated dimerization has minimal influence on protein thermostability. The disruption of salt bridges K118-D177 and D219-R233 decreases T_m_ by 12.3 and 8.6°C, respectively. Interestingly, residues D177 and D219 belong to loop regions of tDGC, suggesting a greater contribution to thermostability of salt bridges located in flexible regions of the protein. In contrast, salt bridge R152-E196, which connects alpha helices α3 and α4 has a more modest influence on thermostability (T_m_ decrease of 4.2°C). In addition to salt-bridges, a contribution to the thermostability of tDGC could derive from a larger number of hydrophobic interactions [35] (**Table 1** in File S1). This aspect was not studied here.

In conclusion, our study identified a dimeric active-like conformation for a thermostable GGDEF domain, where the relative orientation of both active sites is compatible with c-di-GMP formation. The R158A mutation in the I-site abolishes feedback inhibition by preventing formation of the inactive c-di-GMP-locked dimer. Our work also points to a few structural features accounting for enhanced thermostability of tDGC including the formation of three surface exposed unique salt bridges. Point mutations introducing salt bridges in solvent-exposed loops could constitute one possible approach to engineer more thermostable proteins.

## Supporting Information

File S1 **Table 1, Table 2, Figure S1 and Figure S2.**
(DOCX)
