# Supplementary material for: Structure of a Diguanylate Cyclase from Thermotoga maritima: Insights into Activation, Feedback Inhibition and Thermostability
Source: PLoS One. 2014 Oct 31;9(10):e110912. doi: 10.1371/journal.pone.0110912 (PMC4215984; doi:10.1371/journal.pone.0110912)
Supplement: File S1 — Table 1, Table 2, Figure S1 and Figure S2. (DOCX) [file pone.0110912.s001.docx]

**Structure of a diguanylate cyclase from *Thermotoga maritima*: insights into activation, feedback inhibition and thermostability**

Angeline Deepthi^1^, Chong Wai Liew^2^, Zhao-Xun Liang^2^, Kunchithapadam Swaminathan^1, ‡^ and Julien Lescar^2, 3, ‡^

**Supplementary Data**

**(2 supplementary Tables and 2 Supplementary Figures)**

**Supplementary Table 1.** Comparison of structural parameters of tDGC and its homologues.

| Characteristics | PDB code | | | | |
| --- | --- | --- | --- | --- | --- |
|  | tDGC | 3ICL | 4IOB | 3IGN | 3EZU |
| Organism (growth conditions) | *Thermotoga maritima* (Hyper Thermophile) | *Methylococcus capsulatus* (Thermotolerant) | *Pseudomonas aeruginosa* (Mesophile) | *Marinobacter aquaeolei* (Mesophile) | *Geobacter sulfurreducens* (Mesophile) |
| Resolution (Å) | 2.27 | 2.00 | 2.78 | 1.83 | 1.95 |
| Number of Residues | 162 | 163 | 160 | 165 | 159 |
| Sequence Identity (%) | - | 40 | 40 | 35 | 36 |
| RMSD^a^ (number of atoms aligned) | - | 1.34 (153) | 1.34 (155) | 1.73 (159) | 1.46 (144) |
| Number of Main Chain H-bonds^b^ | 124 | 120 | 123 | 130 | 116 |
| Number of side chain H-bonds^b^ | 54 | 62 | 39 | 70 | 62 |
| Relative Contact Order^c^ | 0.105 | 0.107 | 0.106 | 0.107 | 0.110 |
| Surface to Volume Ratio^b^ | 0.39 | 0.378 | 0.40 | 0.40 | 0.40 |
| Total ASA (Å^2^)^b^ | 8836.7 | 7873.9 | 8566.8 | 8692.1 | 7953.5 |
| Total Charged ASA (Å^2^)^b^ | 2660.9 | 1630.3 | 2509.7 | 2349.0 | 1607.6 |
| Total Polar ASA (Å^2^)^b^ | 1259.5 | 1589.1 | 1413.5 | 1802.3 | 1451.7 |
| Total Hydrophobic ASA (Å^2^)^b^ | 4916.3 | 4654.1 | 4643.6 | 4540.8 | 4894.2 |
| Hydrophobic ASA/Total ASA | 0.56 | 0.59 | 0.54 | 0.52 | 0.62 |
| Number of salt bridges (far apart in primary structure)^e^ | 5 | 2 | 2 | 2 | 1 |

1. RMSD were calculated using the iPBA server [[1](#_ENREF_1)]. *b*- ASA: accessible surface area. Parameters calculated using the VADAR server [[2](#_ENREF_2)]. *c*- Relative Contact order calculated using the server at [**http://depts.washington.edu/bakerpg/contact_order/**](http://depts.washington.edu/bakerpg/contact_order/)**.** d- –Salt Bridges were evaluated using the ESBRI[[3](#_ENREF_3)] server. Monomer A was used for all calculations. For structure 3EZU, the C terminal region spanning residues 182 to 341 was used for the calculations.

**Supplementary Table 2.** Salt-bridge interactions in GGDEF domains tDGC, 3ICL, 4IOB, 3IGN and 3EZU.

| PDB ID | RESIDUES | DISTANCE (Å) |
| --- | --- | --- |
| tDGC | K118-D177  R152-E196  R165-D91  R233-D219  K238-D126 | 3.34  3.95  3.53  3.19  3.17  3.64  3.12  3.00  3.86  2.99 |
| 3ICL | R75-D1  K149-D36 | 2.85  3.43  3.80 |
| 4IOB | K401-D287 | 3.96 |
|  | K401-D289 | 3.75 |
| 3IGN | R232-D158 | 3.17  3.45 |
|  | K306-D193 | 2.92 |
| 3EZU | R158-D184 | 3.34  2.92 |

**
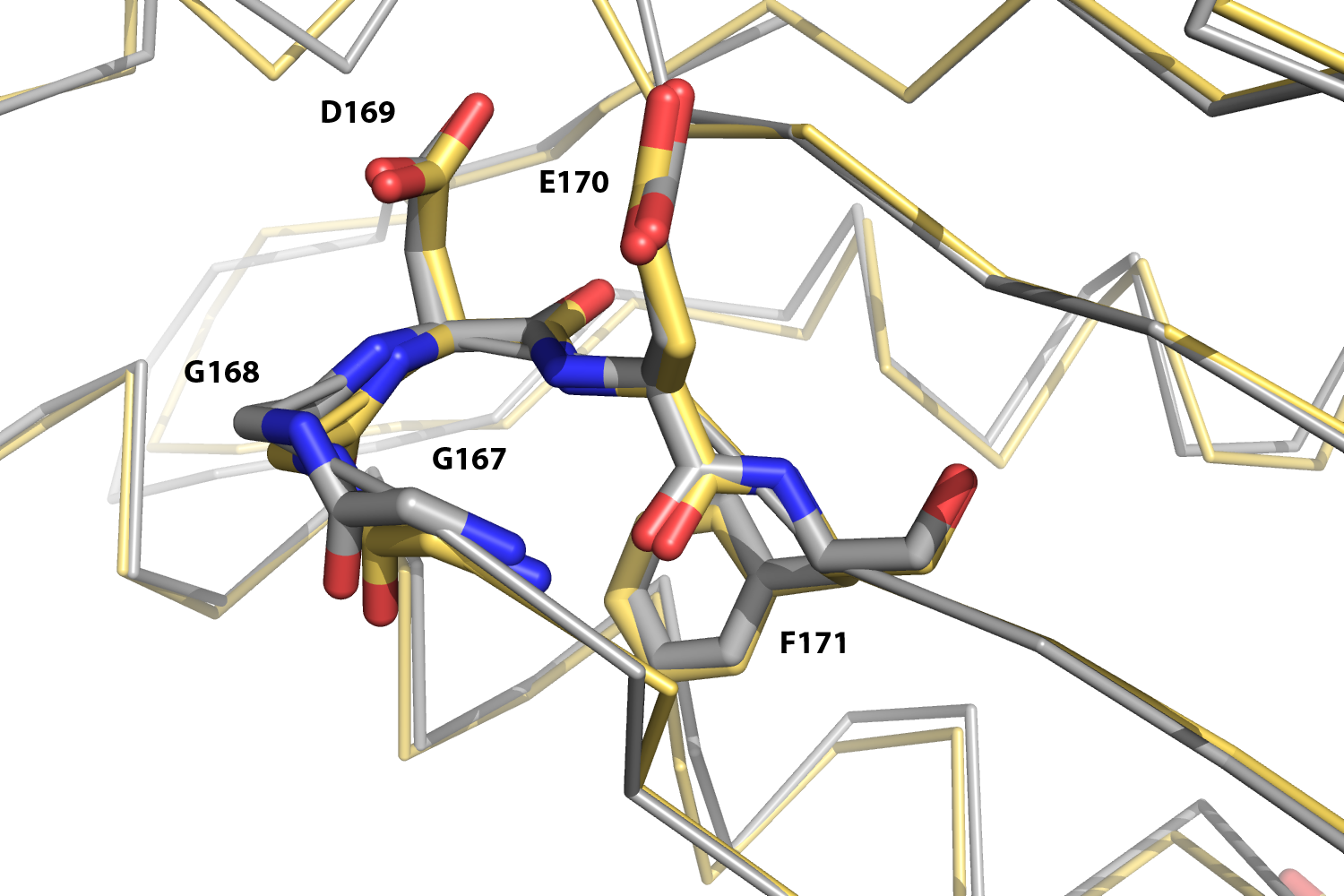
**

**Figure S1** Comparison of A-site residues of tDGC with (yellow) and without (grey) bound c-di-GMP at the I site. The conformations of the main-chain and side-chains are closely similar indicating an absence of signal relay from the I-site to the A-site of the protein upon c-di-GMP binding.


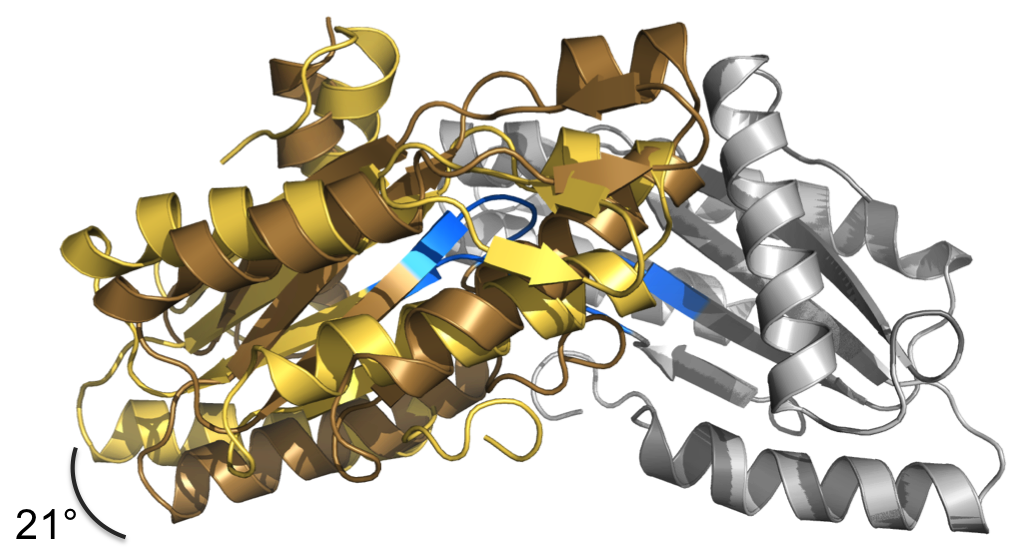


**Figure S2** Superposition of the “active-like dimer” (colored in brown) that was crystallized and the deduced (“optimized”) catalytically active dimer (colored in yellow). The r.m.s.d. between monomers colored in yellow and brown is 0.79 Å (following superposition of the grey monomer). The transformation that must be applied to generate a symmetric monomer with exact 2-fold rotation symmetry requires an additional rotation of 21 degrees.

**References**

1. Gelly JC, Joseph AP, Srinivasan N, de Brevern AG (2011) iPBA: a tool for protein structure comparison using sequence alignment strategies. Nucleic Acids Res 39: W18-23.

2. Willard L, Ranjan A, Zhang H, Monzavi H, Boyko RF, et al. (2003) VADAR: a web server for quantitative evaluation of protein structure quality. Nucleic Acids Res 31: 3316-3319.

3. Costantini S, Colonna G, Facchiano AM (2008) ESBRI: a web server for evaluating salt bridges in proteins. Bioinformation 3: 137-138.
